# Construction of warfarin population pharmacokinetics and pharmacodynamics model in Han population based on Bayesian method

**DOI:** 10.1038/s41598-024-65048-7

**Published:** 2024-06-27

**Authors:** Xiaotong Xia, Xiaofang Cai, Jiana Chen, Shaojun Jiang, Jinhua Zhang

**Affiliations:** Department of Pharmacy, Fujian Maternity and Child Health Hospital College of Clinical Medicine for Obstetrics and Gynecology and Pediatrics, Fujian Medical University, #18 Daoshan Road, Fuzhou, 350001 China

**Keywords:** Population PK/PD model, Warfarin, Gene polymorphism, Han population, Cardiology, Diseases, Medical research

## Abstract

The purpose of this paper is to study the genetic polymorphisms of related gene loci (CYP2C9*3, VKORC1-1639G > A) based on demographic and clinical factors, and use the maximum a posterior Bayesian method to construct a warfarin individualized dose prediction model in line with the Chinese Han population. Finally, the built model is compared and analyzed with the widely used models at home and abroad. In this study, a total of 5467 INR measurements are collected from 646 eligible subjects in our hospital, and the maximum a posterior Bayesian method is used to construct a warfarin dose prediction that conforms to the Chinese Han population on the basis of the Hamberg model. The model is verified and compared with foreign models. This study finds that body weight and concomitant use of amiodarone have a significant effect on the anticoagulant effect of warfarin. The model can provide an effective basis for individualized and rational dosing of warfarin in Han population more accurately. In the performance of comparison with different warfarin dose prediction models, the new model has the highest prediction accuracy, and the prediction percentage is as high as 72.56%. The dose predicted by the Huang model is the closest to the actual dose of warfarin. The population pharmacokinetics and pharmacodynamics model established in this study can better reflect the distribution characteristics of INR values after warfarin administration in the Han population, and performs better than the models reported in the literature.

## Introduction

Warfarin is a coumarin oral anticoagulant and a vitamin K antagonist. It is used in the treatment of valvular disease, post-valvular valve replacement, non-valvular atrial fibrillation and deep vein thrombosis^1,2^. According to statistics, millions of patients worldwide are using warfarin^3^. There are many factors that influence the efficacy of warfarin, including genetic factors and non-genetic factors. Currently, there are two main methods for developing warfarin individualized dosing models: multiple regression analysis (MRA)^3^ and maximum a posterior Bayesian (MAPB)^4^. Numerous MRA prediction models for warfarin doses have been published at home and abroad, but most of these models only predict the maintenance dose or initial dose, and only have a prediction accuracy of about 37–55%^5^. At present, the widely used dose and INR prediction model at home and abroad is the Hamberg model with Caucasians as the research group^6^. However, due to the large dose difference between patients of different ethnic groups^7^, there is still room for improvement in the accuracy for this type of model used in the Han population. Although the model has been validated abroad^8^, there are no reports in the literature that use the Chinese population as a sample. In this part of the study, an individualized PPK/PD and INR prediction model will be constructed and named as the Alfalfa-Warfarin-PPK/PD model. The model can be used to calculate the INR value or dose at any time, providing a basis for individualized precision medicine for Chinese patients taking warfarin. Especially for patients with long-term anticoagulation therapy, the INR value before the next follow-up can be predicted, which has good clinical application value.

## Method

### Research objects

The subjects of the study are patients from Fujian Medical University Union Hospital and 8 other sub-central hospitals from March 2016 to October 2020. All selected patients have signed the informed consent. This study has been approved by the hospital ethics committee (2016KY036, 2020KY006). And all methods were performed in accordance with the relevant guidelines and regulations. Relevant demographic information (age, sex, height, and weight) and concomitant medications (amiodarone, statins, azole antifungals, and broad-spectrum antibiotics) have been collected.

Inclusion criteria:

1. The basal INR value is measured before taking the medicine;
2. Take warfarin regularly (take the medicine at a fixed time every day without missing a dose);
3. The detected INR value is recorded with the specific measurement time;

Exclusion criteria:

1. Malignant tumor;
2. Severe mental illness or mental disorder;
3. Pregnancy or lactation;
4. Severe renal insufficiency (endogenous creatinine clearance rate less than 15 mL/min, serum creatinine ≥ 200 μmol/L^7^);
5. Severe hepatic insufficiency (bilirubin is higher than 2 times the upper limit of normal, and Aspartate Transaminase (AST)/Alanine Aminotransferase (ALT)/alkaline phosphatase is higher than 3 times the upper limit of normal^9^).

### Pharmacodynamic analysis of population pharmacokinetics

Hamberg’s model is currently widely used for the individualized use of warfarin, This article will estimate model parameters applicable to Han Chinese populations based on this model (set as a reference literature model)^10^ and based on extensive Chinese population data (646 subjects from 9 centers in different regions of China with 8352 drug records and 5467 INR measurements). The methodology for the construction and evaluation of the model is described in the supplementary material.

### Data analysis

NONMEM (Version 7.4, ICON Development Solutions, USA) is used to establish population pharmacokinetic and pharmacodynamic model and model simulation; the plug-in of the running interface is Wings for NONMEM; R software (Version 3.6.3) is used for drawing. In covariate screening, the covariates is firstly investigated and the model parameter EC50 values are analyzed by the graphical method, and the significantly correlated covariates were screened (*P* < 0.05). In addition to CYP2C9 genotype, VKORC1 genotype and age, this study will also examine factors that effect on model parameter EC50 values, such as body weight, body mass index, gender, concomitant use of amiodarone, azole antibiotics, statins and broad-spectrum antibiotics. Model evaluation uses the Bootstrap method to perform 1000 replaceable repeated sampling to obtain the median and 95% confidence interval of the model parameter distribution, and compare them with the model parameters obtained from the original data set, so as to evaluate the stability of the model parameters.

### Ethical standards

All selected patients have signed the informed consent. This study has been approved by the hospital ethics committee (2016KY036, 2020KY006).

## Results

### Basic clinical information

A total of 5467 INR measurements are collected from 646 subjects. The daily dose of warfarin ranges from 0.625 to 6.25 mg, and the frequency of administration is once a day. The time of INR measurement is distributed within 24 h after administration, mainly in 7–10 h after administration.

In this study, the data of subjects with ID of 1–114 (a total of 109 subjects with 969 INR measurements) are designated as the validation data set, and the data of subjects with ID of 115-1103 (a total of 537 subjects) are designated as validation data sets (in total of 537 subjects and 4498 INR measurements. The general characteristics of the subjects are detailed in Table 1.

**Table 1:** General data analysis (measurement data).

	Modeling dataset N = 537	Validation dataset N = 109	Total data N = 646
Age (year)			
Mean ± SD	55.2 ± 12.5	52.3 ± 12	54.7 ± 12.5
Min–Max	1–82	18–72	1–82
95% CI	26–76	27–70.3	27–76
Height (cm)			
Mean ± SD	161.7 ± 9.5	162.9 ± 8.6	161.9 ± 9.3
Min–Max	80–197	141–188	80–197
95% CI	145–178	143.8–182	145–179.7
Weight (kg)			
Mean ± SD	59.5 ± 11.6	60.7 ± 11.5	59.7 ± 11.6
Min–Max	7–106	36.5–102	7–106
95% CI	38.5–85	38.8–85.9	39–85
BMI (kg/m^2^)			
Mean ± SD	22.7 ± 3.7	22.8 ± 3.5	22.7 ± 3.6
Min–Max	12.5–35.8	14.5–34.5	12.5–35.8
95% CI	15.8–30	17.1–30.1	16.1–30
INR baseline			
Mean ± SD	1.14 ± 0.64	1.08 ± 0.28	1.13 ± 0.59
Min–Max	0.82–12.79	0.84–3.66	0.82–12.79
95% CI	0.85–2.31	0.88–1.6	0.85–2.25
Sex			
Male	270 (50.3%)	60 (55%)	330 (51.1%)
Female	267 (49.7%)	49 (45%)	316 (48.9%)
*CYP2C9*			
*1/*1	506 (94.2%)	103 (94.5%)	609 (94.3%)
*1/*3	31 (5.8%)	6 (5.5%)	37 (5.7%)
*VKORC1*			
*AA*	427 (79.5%)	92 (84.4%)	519 (80.3%)
GA	106 (19.7%)	15 (13.8%)	121 (18.7%)
*GG*	4 (0.7%)	2 (1.8%)	6 (0.9%)
Amiodarone			
Yes	129 (24%)	37 (33.9%)	166 (25.7%)
No	408 (76%)	72 (66.1%)	480 (74.3%)
Azole antibacterials			
Yes	8 (1.5%)	9 (8.3%)	17 (2.6%)
No	529 (98.5%)	100 (91.7%)	629 (97.4%)
Statins			
Yes	153 (28.5%)	88 (80.7%)	241 (37.3%)
No	384 (71.5%)	21 (19.3%)	405 (62.7%)
Antibiotics			
Yes	3 (0.6%)	1 (0.9%)	4 (0.6%)
No	534 (99.4%)	108 (99.1%)	642 (99.4%)

### Alfalfa-Warfarin-PPK/PD model and evaluation

**Figure 1:**
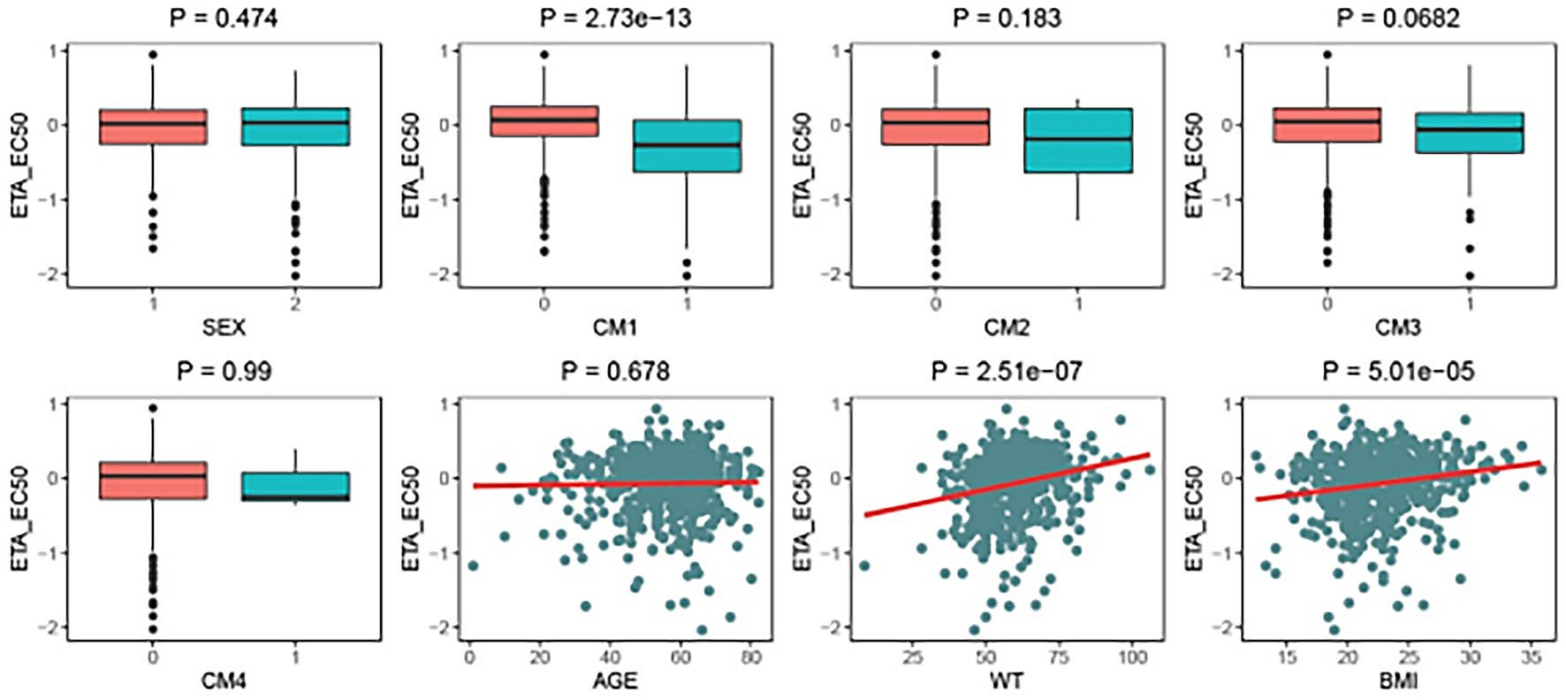
Correlation analysis of covariates and model parameters EC50 inter-individual variation (ηEC50). (SEX: Sex, 1 for male, 2 for female; CM1: whether amiodarone, 0 unsuitable, 1 combined; CM2: azole antibiotics, 0 useless, 1 combined; CM3, statin, 0 useless, 1 combined; CM4: broad spectrum antimicrobial, 0 unsuitable, 1 combined; AGE: Age; WT: body weight; BMI: Body mass index).

The correlation analysis shows that the inter-individual variation value (ηEC_50_) of the model parameter EC_50_ is significantly correlated with body weight, body mass index and amiodarone (*P* < 0.05), while the correlation between other covariates and the model parameters is not significant (*P* > 0.05) (Fig. 1). It is manifested as a significant decrease in the EC_50_ value when amiodarone is combined, that is, the effect value of anticoagulation increases under the same warfarin dose; When body weight or body mass index increases, the EC_50_ value increases, that is, the anticoagulant effect value decreases at the same warfarin dose.

The graphical correlation analysis shows that the inter-individual variation value of model parameter EC50 (ηEC50) has a more significant correlation with body weight, and it is easier to consider the clinical use of body weight. Therefore, this study finally selects body weight to be included in the covariate analysis (Table 2).

**Table 2:** Covariate model building process.

Model number	Model	OFV	− 2LL
Base model		574.218	
First round forward introduction			
1	Base model + CM1 on EC_50_	536.65	− 37.56
2	Base model + WT on EC_50_	411.269	− 162.949
Second round forward introduction			
3	Model 2 + CM1 on EC_50_	366.848	− 444.421
The first round of reverse culling			
4	Model 3 − WT on EC_50_	536.65	169.802
5	Model 3 − CM1 on EC_50_	411.269	44.421

CM1: whether amiodarone is used in combination; WT: body weight.

In addition to the covariates included in the base model, the newly added covariate model of the reconstructed model is expressed as follows: EC50 = TVEC50 × (WT/60)1.34-CM1 × 0.602.

In the formula, WT is body weight, CM1 is whether amiodarone is used in combination, 1 represents combined use, and 0 represents no combination. The TVEC50 value is a typical value of the EC50 value, and the TVEC50 value of different VKORC1 genotypes is different. For details, please refer to the methodological covariate model construction section. The model results show that when amiodarone is used in combination, the EC50 value of the anticoagulant effect will decrease by 0.602 mg/L, and the EC50 value of the anticoagulant effect will increase by 22.9% for every 10 kg increase in body weight.

1000 times Bootstrap repeated sampling method is used for internal verification, of which 845 times parameters are successfully estimated, and the results are analyzed. The results show (Table 3) that the 95% confidence interval of the newly added covariate parameters in the Alfalfa-Warfarin-PPK/PD model does not include 0, indicating that the newly added covariates have a significant impact on EC50 and are not affected by the sample, so the Alfalfa-Warfarin-PPK/PD model retains the above covariates. In addition, the median parameter estimation obtained by Bootstrap is basically consistent with the parameter estimation value of the original data set, which reflects the stability of the model parameter estimation.

**Table 3:** Final model parameter estimates.

	Final model estimate (relative standard error %)	Bootstrap validation Median (2.5–97.5% quantile)
PK parameter		
CL per *1 allele (L/h)	0.174, Fixed	–
CL per *2 allele (L/h)	0.0879, Fixed	–
CL per *3 allele (L/h)	0.0422, Fixed	–
Effect of *ag*e (% change/year) on CL	− 0.571, Fixed	–
V/F, L	14.3, Fixed	–
PD parameter		
E_max_	0.174, Fixed	–
γ	1.39, Fixed	–
EC_50_ per *VKORC1* G allele (mg/L)	4.3 (16.3)	4.39 (3.18, 5.84)
EC_50_ per *VKORC1* A allele (mg/L)	1.14 (7.7)	1.16 (0.99, 1.328)
WT on EC_50_	1.34 (34.3)	1.45 (0.724, 2.14)
CM1 on EC_50_	− 0.602 (46.7)	− 0.584 (− 0.991, − 0.089)
MTT_1_ (h)	27.2, Fixed	–
MTT_2_–MTT_1_ (h)	83.7, Fixed	–
MTT_2_ (h)	110.9, Fixed	–
INR_max_	20, Fixed	–
Inter-individual variation		
η(EC_50_), %	55.8 (8.2)	56.7 (49.3, 66.7)
Intra-individual variation		
ε_prop_, %	36.5 (4.1)	37.0 (33.8, 40.2)

MTT1: Average transfer time for transfer room 1.

MTT2: Average transfer time for transfer room 2.

1000 Bootstrap has 845 minimization successes. CL values for alleles 1, 2 and 3 are the clearance rates of VKORC1 alleles *1*2*3.

The goodness-of-fit graph (Supplementary Material Figs. 2 and 3) shows that, compared with the basic model, the Alfalfa-Warfarin-PPK/PD model has a certain degree of improvement in the goodness-of-fit to the data, and the outliers of CWRES are slightly reduced compared to the basic model.

### External Verification of Alfalfa-Warfarin-PPK/PD Model

The model parameters in the established Alfalfa-Warfarin-PPK/PD model and the literature model^11^ are fixed, and the INR in the validation dataset is predicted and compared with the measured value to evaluate the extrapolation performance of the two models.

The results show that both reconstructed Alfalfa-Warfarin-PPK/PD model and the literature model have good predictive ability for the INR data in the validation dataset, and the predicted value is very close to the measured value distribution (Supplementary Material Fig. 4). The OFV calculated by the literature model for the verification data set is 15.288, while the OFV calculated by the Alfalfa-Warfarin-PPK/PD model for the verification data set is − 238.464, which is far lower than the former, indicating that the prediction of the Alfalfa-Warfarin-PPK/PD model is better on INR in the validation dataset. In addition, it can be seen from the model diagnostic diagrams (Supplementary Material Figs. 5 and 6) that the CWRES outliers (> 6) under the Alfalfa-Warfarin-PPK/PD model are significantly less than the literature model, which also suggests that the Alfalfa-Warfarin-PPK/PD model performance is better.

## Comparison of Alfalfa-Warfarin-PPK/PD model and other multiple prediction models

At present, there are many warfarin dose prediction models established based on gene polymorphisms, which can better improve the accuracy of individualized warfarin medication. However, there are not many evaluations of its efficacy, especially less evaluation of the predicted efficacy of different stable dose components. The reconstructed warfarin dose prediction model is verified and evaluated, and its performance is compared with various prediction models recognized at home and abroad, so as to evaluate which models have better performance and are more suitable for Han population. A representative model is selected based on the number of people, race, relevant inclusion factors, and whether prospective validation performed in the dose prediction models. These prediction models are used to predict the stable dose of warfarin for the whole group of patients, and the prediction percentage and the mean of absolute mean of error (MPE) are used to evaluate the efficacy of the warfarin dose prediction model.

### Selected research models

Because many hospitals in China use the Gage model and IWPC model for the detection of warfarin VKORC1 and CYP2C9 gene polymorphisms, and guide medication according to the FDA's warfarin dose recommendation table. The previous part of the Alfalfa-Warfarin-PPK/PD model is built on the basis of the Hamberg model. Finally, IWPC dose model, Gage dose model, FDA warfarin application guidance, Hamberg model and Alfalfa-Warfarin-PPK/PD model are selected for the comparison and evaluation of predictive efficacy. Details of the included models can be found in Supplementary Material Table 1.

### Comparison of the prediction dose of warfarin between the Alfalfa-Warfarin-PPK/PD model and other models

**Figure 2:**
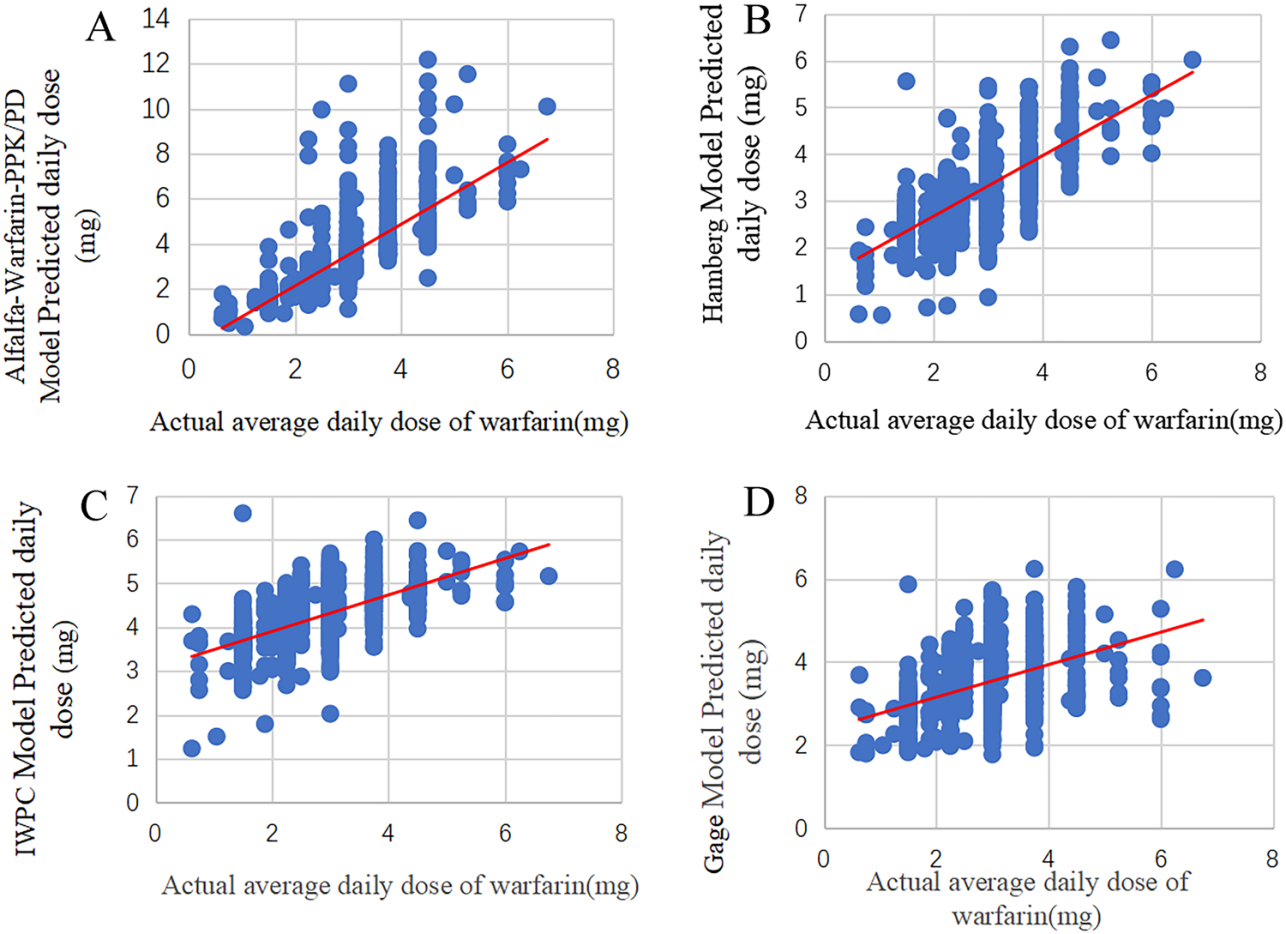
The correlation between the actual average daily dose of warfarin and the prediction dose of the prediction model (Dots represent: warfarin doses; Solid lines: represent regression curves).

The warfarin dose is calculated by the warfarin model formula, and the prediction dose and the actual dose of warfarin are plotted as a scatter plot to obtain the Pearson coefficient. The results show that there is a correlation between prediction dose and actual dose in each model (*P* < 0.001), and they are evenly distributed on both sides of the regression line. See Fig. 2 and Supplementary Material Table 2.

#### Results of the mean absolute error of each model

In this part of the study, the models with MPE values less than the minimum divided dose of warfarin of 0.625 mg^12^ includes the Alfalfa-Warfarin-PPK/PD model and the Hamberg model, which are 0.390 mg/day and 0.427 mg/day, as shown in Supplementary Material Table 3.

#### Results of prediction percentage of each model

The model with the highest ideal prediction percentage is the Alfalfa-Warfarin-PPK/PD model, followed by the Hamberg model. These two models are 72.60% and 51.15% respectively, as shown in Supplementary Material Table 4.

## Discussion

This part of the study is based on the model published by Hamberg, based on extensive data of Han population, combined with the maximum a posterior Bayesian method, to establish a warfarin PPK/PD model suitable for Han population. It can not only predict the initial dose of warfarin like the model constructed by the traditional MAR method, but also can reach the followings: (1) predict the INR value under a given dose maintenance; (2) realize the dose and INR value in an unsteady state, achieve the target INR value as soon as possible, and avoid excessive anticoagulation or insufficient anticoagulation.

### Effect of CYP2C9 gene polymorphism on warfarin PK

The studies by Ma et al. confirm that CYP2C9 gene polymorphism can explain approximately 5–10% of the interindividual warfarin variation^13^; the studies by Hamberg et al. show that the clearance difference between different CYP2C9 genotypes is as high as 4.2-fold^14^. Several studies show that CYP2C9 gene polymorphisms can affect the pharmacokinetic parameters of warfarin, and they are included in the model parameters when constructing a warfarin PK/PD model^13–15^. Several studies show that the CYP2C9 gene polymorphism significantly affects the clearance rate of warfarin, and the *2 or *3 genotype are the main genetic risk factor for excessive anticoagulation and bleeding events of warfarin^16–18^. The results of this study shows that the CYP2C9 *1/*1 genotype has the highest clearance rate of warfarin, followed by *1/*2, *1/*3, *2/*2, *2or*3, CYP2C9 *3/ *3 Genotype has the lowest clearance rate of warfarin. Incorporating the CYP2C9 genotype into the model parameters will more accurately predict the dose of warfarin, avoid excessive anticoagulation, and reduce the occurrence of bleeding events.

### The effect of VKORC1 gene polymorphism on warfarin PD

The studies by Ma et al. confirm that VKORC1 gene polymorphism can explain about 20–25% of the interindividual warfarin variation^14^, and the studies by Hamberg et al.^11^ show that it’s up to twofold difference in EC50 between different VKORC1 genotypes. Several studies also show that VKORC1 genotype polymorphism can affect the pharmacodynamics of warfarin, and it is included as a model parameter in the construction of warfarin PPK/PD models^14–16^. Several studies show that the VKORC1-1639 genotype polymorphism affects the anticoagulant effect of warfarin, and is significantly associated with excessive anticoagulation and bleeding events in the primary phase of warfarin use^14,18,19^. This article shows that the VKORC1-1639 genotype polymorphism has a strong correlation with the model parameter EC50 value (*P* < 0.05), among which the VKORC1-1639GG genotype has the highest EC50 value (9.30 mL/min), VKORC1-1639GA genotype is the second (5.64 mL/min), and VKORC1-1639AA is the smallest (1.98 mL/min). Therefore, VKORC1-1639 is included in the model parameters. For patients with genetic variation, if they can understand their genetic information before or in the early stage of medication, predicting the dose according to the model formula can not only help clinicians or pharmacists understand the dose required by patients as soon as possible, but also shorten the time to reach the target INR, which can also reduce the number of repeated blood draws, reduce costs, and increase patient compliance.

### The effect of age on warfarin PK

Several studies show that although the protein binding capacity of warfarin does not change with age, its clearance rate in the body decreases with age, and the efficacy of warfarin gradually increases^20–26^. The studies of Vear et al. show that genetic polymorphisms affect the pharmacokinetics and pharmacodynamics of warfarin, and the effect of genetic polymorphisms increases with age^27^. In this study, age is introduced into the model parameter CL, and the results show that the value of CL decreased by 0.571% for every 1-year increase in age. Therefore, when using warfarin clinically, the effect of age on the stable dose of warfarin should also be considered.

### Effects of body weight and gender on warfarin PD

Relevant studies show that when patients with BMI ≥ 40 kg/m^2^ (or body weight > 120 kg) and body weight < 50 kg use oral anticoagulants, drug distribution and metabolism will be significantly affected^28,29^. Studies by Lane^26^ and Xu^30^ also show that patient weight can significantly affect S-warfarin clearance, affect the pharmacokinetics of warfarin, and thus affect pharmacodynamics. This study shows that when body weight or body mass index increases, the EC50 value increases. That is, under the same warfarin dose, the effect value of anticoagulation decreases. When the body weight increases by 10 kg, the EC50 value will increase by 22.9%.

This part of the study analyzes the correlation between gender and model parameter EC50 value. The *P* > 0.05, which indicates that gender difference has no significant effect on the PD of warfarin. The studies by Hamberg and Lin Rongfang et al. also show that age has no significant effect on the model parameter EC50 in the study of warfarin PK/PD model, and has not been included in the model construction.

### Effect of concomitant medication on warfarin PD

For warfarin combined with amiodarone, several studies demonstrate that amiodarone has a significant effect on the anticoagulant effect of warfarin^31,32^. In this study, it shows that when patients are combined with amiodarone, the EC50 value of the anticoagulant effect will decrease by 0.602 mg/L. So to achieve the same anticoagulant effect, patients combined with amiodarone must reduce the dose of warfarin.

For the relationship between azole antifungals and warfarin PD, related studies prove that azole antifungals are substrates and inhibitors of cytochrome P450 isoenzymes^33^, and the metabolism of warfarin needs to pass through P450 enzymes. So when warfarin is combined with azole antifungals, the anticoagulant effect of warfarin is enhanced to some extent. The results of this study show that azole antifungal drugs have no effect on the EC50 of warfarin. On the one hand, it may be that the patients included in this study have fewer azole antifungal drugs. On the other hand, more than 50% of the patients taking azole antifungal drugs may have used itraconazole, which has no inhibitory effect on CYP2C9. Most of the remaining patients use fluconazole and voriconazole with weaker inhibitory effects, so the overall sample has a small impact on the anticoagulant effect of warfarin.

For the association of statins with warfarin PD, a number of studies show that fluvastatin can significantly increase the anticoagulation intensity of warfarin, atorvastatin does not affect the anticoagulation intensity of warfarin, and there are large individual differences in the effect of rosuvastatin on the anticoagulation intensity of warfarin. In this study, the model parameter EC50 of statins and warfarin show little correlation, which may be because the patients included in this study who take atorvastatin without affecting anticoagulation intensity accounted for more than 70% of the total patients taking statins. The overall sample has no effect on the anticoagulant effect of warfarin. However, with the increasing number of patients with cardiovascular disease and more and more cases of warfarin combined with statin, it is still necessary to strengthen the monitoring of INR value and bleeding risk when the two drugs are combined.

For the impact of broad-spectrum antibiotics on warfarin PD, some studies confirm that broad-spectrum antibiotics can affect the anticoagulant effect of warfarin^34,35^. Several studies show that chloramphenicol, cefazolins, cefoperazone, cefotaxime, and erythromycin can reduce the absorption of vitamin K. Combination with warfarin can prolong the synthesis time of prothrombin, making warfarin anticoagulant enhanced. Metronidazole, chloramphenicol, etc. can inhibit the CYP enzyme activity in the liver, thereby reducing the clearance rate of warfarin and enhancing its anticoagulant effect. In this study, it shows that the model parameter EC50 of broad-spectrum antibiotics and warfarin has little correlation, which may be because the patients taking roxithromycin and azithromycin have no significant effect on the anticoagulant effect of warfarin, accounting for about 50% of the total patients taking antibiotics.

### Evaluation of the predictive performance of each research model

There is a certain correlation between the prediction dose of the five models and the actual clinical dose (*P* < 0.001), and the models with a strong positive correlation between the prediction dose and the actual dose (r > 0.07) are the Alfalfa-Warfarin-PPK/PD model and the Hamberg model, which are 0.767 and 0.720 respectively, among which the Alfalfa-Warfarin-PPK/PD model has a stronger correlation. The prediction dose and actual dose of the remaining models are moderately correlated (r = 0.04–0.07). In the comparison of the mean prediction error, the model whose MPE value less than the minimum divided dose of warfarin (0.625 mg/day) is the Alfalfa-Warfarin-PPK/PD model and the Hamberg model, indicating that the prediction dose of these two models is close, compared with the actual stable dose of the patient. The prediction dose of the Alfalfa-warfarin-PPK/PD model is closest to the actual dose of warfarin.

In the prediction percentage analysis, the higher prediction percentage the model overestimates, the higher probability of the prediction dose calculated by the selected model is higher than the actual dose, and the greater the risk of bleeding will be in patient taking the prediction dose^36^. The highest ideal prediction percentage in this study is the Alfalfa-Warfarin-PPK/PD model, which is as high as 72.6%, indicating that among these models, the risk of thrombosis and hemorrhage is the lowest using prediction warfarin dose by the Alfalfa-Warfarin-PPK/PD model, followed by the Hamberg model. The percentage of underestimated predictions is low for all models, and the percentage of overestimated predictions is higher except for the Alfalfa-Warfarin-PPK/PD model, indicating that these models are more likely to overpredict patients actual dose and the risk of bleeding in patients dosed as predicted by these models may be higher than the risk of thrombosis. This may be because the distribution frequency of gene polymorphisms in the Chinese population is different from other ethnic groups. The frequency of CYP2C9*3 gene distribution in the sample of this study is only 4.16%. Several studies show that the frequency distribution of CYP2C9*3 gene in the Chinese population is relatively low, which is about 3.5%, and up to 18% in other ethnic groups^37,38^. The frequency distribution of the VKORC1-1639AA genotype in the sample is as high as 92.03%, and related studies show that the distribution frequency of the VKORC1-1639AA genotype in the Asian population is significantly higher than that in the Caucasian population, with a distribution frequency of about 80.4%, but the frequency distribution in the Caucasian population is less than 50% of the total population^39,40^. Most of the above models are based on European populations. For example, the FDA warfarin dosage guidelines, Gage model and Hamberg model are all based on European and American populations. Most of the populations in the WICP model are also European and American populations.

Compared with other Chinese population models, our study includes patients in a larger number of centers, and is not limited to patients after heart valve replacement. In the study of Zhu et al., a new population pharmacokinetic model is established and individual apparent clearance is obtained^41^. Together with other influencing factors, a prediction model of patient maintenance dose is constructed by multiple linear regression method. Our PK is directly fixed as the parameter estimation value of the literature model, and the EC50 value of the model parameter of PD is based on a wide range of Chinese Han population data, combined with the maximum posterior Bayes method, and re-estimated according to the data characteristics of Chinese Han population. Not only can the initial dose of warfarin be formulated, but the maintenance dose of warfarin can also be predicted “point-to-point” through Bayesian feedback. The shortcomings in this study are as follows: The effects of other concomitant drugs on the pharmacokinetics of warfarin has not been considered, and only the effects of statins, broad-spectrum antimicrobials, azole antifungals, and amiodarone on the pharmacokinetics of warfarin, which have a large number of concomitant patients, has been analyzed. However, in clinical practice, some patients will combine other drugs that may interact with warfarin, such as antihypertensive drugs, hypoglycemic drugs and traditional Chinese medicines, which may affect the pharmacokinetics of warfarin. In the process of data verification in this study, it has not been completely randomized, and when evaluating the efficacy of each warfarin model, no prospective intervention has been carried out. If prospective intervention could be carried out, it could also evaluate the standard time and adverse reactions of patients according to the model prediction of drug administration and routine administration, which can better guide clinical medication.

This study also shows that the R^2^ value in the dose model formula is quite different from the prediction accuracy of the model. The studies by Shin et al. also show that the R^2^ value during modeling and whether the model has been internally verified are irrelevant with the model's prediction of warfarin dose^42^.

## Conclusion

This study is the first time to use the Chinese as a sample to conduct an broader study of warfarin PPK/PD model and INR prediction mode.The PPK model is based on Hamberg's model and uses a wide range of Chinese population data to estimate PPK model parameters applicable to Chinese Han population. Compared with the widely used Hamberg model, Alfalfa-warfarin-PPK/PD model includes two more covariates (body weight and amiodarone), and has better goodness of fit, stronger stability, and better predictive performance. It can achieve dose and the mutual calculation of INR values, and better predict the distribution characteristics of INR values after warfarin administration in the Chinese population.

Compared with other models, the prediction dose of the Alfalfa-Warfarin-PPK/PD model is the closest to the actual dose (MPA = 0.39 mg/day), and the ideal prediction percentage is the highest (72.56%). It shows that when the prediction dose of this model is used, the probability of bleeding or thrombosis is the lowest when compared with that of other models, and it has better clinical application value.

### Supplementary Information

Supplementary Information.

## Data Availability

The data that support the findings of this study are available from the corresponding author upon reasonable request.

## References

[1] Crader MF, Johns T, Arnold JK (2020). Warfarin Drug Interactions.

[2] Kearon C, Akl EA, Ornelas J, Blaivas A, Jimenez D, Bounameaux H, Huisman M, King CS, Morris TA, Sood N, Stevens SM, Vintch JRE, Wells P, Woller SC, Moores L (2016). Antithrombotic therapy for VTE disease: Chest guideline and expert panel report. Chest.

[3] Lin M, Zhang J, Yu L, Song H (2014). Advances of individualized administration model of warfarin based on pharmacogenomics. Chin. J. Clin. Pharmacol. Ther..

[4] Tan D, Yan H, Luo Z (2015). Research progress of establishing the gene-guided dosing predictive model of warfarin. Chin. J. Clin. Pharmacol. Ther..

[5] Lubitz SA, Scott SA, Rothlauf EB (2010). Comparative performance of gene-based warfarin dosing algorithms in a multiethnic population. J. Thromb. Haemost..

[6] Dong J, Shi GH, Lu M (2019). Evaluation of the predictive performance of bayesian dosing for warfarin in Chinese patients. Pharmacogenomics.

[7] Lenzini P, Wadelius M, Kimmel S (2010). Integration of genetic, clinical, and INR data to refine warfarin dosing. Clin. Pharmacol. Ther..

[8] Hamberg AK, Hellman J, Dahlberg J, Jonsson EN, Wadelius M (2015). A Bayesian decision support tool for efficient dose individualization of warfarin in adults and children. BMC Med. Inform. Decis. Mak..

[9] Wright DF, Duffull SB (2013). A Bayesian dose-individualization method for warfarin. Clin. Pharmacokinet..

[10] Andrus MR (2004). Oral anticoagulant drug interactions with statins: Case report of fluvastatin and review of the literature. Pharmacotherapy.

[11] Hamberg AK, Wadelius M, Lindh JD (2010). A pharmacometric model describing the relationship between warfarin dose and INR response with respect to variations in CYP2C9, VKORC1, and age. Clin. Pharmacol. Ther..

[12] Wen-feng LIU, Kun-lin JIA, Cui-rong XU (2021). Analysis of the guiding value of CYP2C9 and VKORC1 gene testing for individualized warfarin anticoagulant therapy in patients with acute pulmonary thromboembolism. J. Clin. Exp. Med..

[13] Hamberg AK, Dahl ML, Barban M (2007). A PK–PD model for predicting the impact of age, CYP2C9, and VKORC1 genotype on individualization of warfarin therapy. Clin. Pharmacol. Ther..

[14] Ma Z, Cheng G, Wang P (2019). Clinical model for predicting warfarin sensitivity. Sci. Rep..

[15] Lin M, Yu L, Qiu H (2016). Verification of five pharmacogenomics-based warfarin administration models. Indian J. Pharmacol..

[16] Sasaki T, Tabuchi H, Higuchi S (2009). Warfarin-dosing algorithm based on a population pharmacokinetic/pharmacodynamic model combined with Bayesian forecasting. Pharmacogenomics.

[17] Wright DF, Duffull SB (2011). Development of a Bayesian forecasting method for warfarin dose individualization. Pharm. Res..

[18] Anderson JL, Horne BD, Stevens SM (2007). Randomized trial of genotype-guided versus standard warfarin dosing in patients initiating oral anticoagulation. Circulation.

[19] Gaikwad T, Ghosh K, Kulkarni B (2013). Influence of CYP2C9 and VKORC1 gene polymorphisms on warfarin dosage, over anticoagulation and other adverse outcomes in Indian population. Eur. J. Pharmacol..

[20] Lund K, Gaffney D, Spooner R (2012). Polymorphisms in VKORC1 have more impact than CYP2C9 polymorphisms on early warfarin international normalized ratio control and bleeding rates. Br. J. Haematol..

[21] Khoury G, Sheikh-Taha M (2014). Effect of age and sex on warfarin dosing. Clin. Pharmacol..

[22] Garcia D, Regan S, Crowther M (2005). Warfarin maintenance dosing patterns in clinical practice: Implications for safer anticoagulation in the elderly population. Chest.

[23] Khaleqsefat E, Khalaj-Kondori M, Jabarpour Bonyadi M (2020). The contribution of VKORC1 and CYP2C9 genetic polymorphisms and patients' demographic characteristics with warfarin maintenance doses: A suggested warfarin dosing algorithm. Iran. J. Pharm. Res..

[24] Pathare A, Khabori MA, Alkindi S (2012). Warfarin pharmacogenetics: Development of a dosing algorithm for Omani patients. J. Hum. Genet..

[25] Wei M, Ye F, Xie D (2012). A new algorithm to predict warfarin dose from polymorphisms of CYP4F2, CYP2C9 and VKORC1 and clinical variables: Derivation in han chinese patients with non valvular atrial fibrillation. Thromb. Haemost..

[26] Jensen BP, Chin PK, Roberts RL (2012). Influence of adult age on the total and free clearance and protein binding of (R)- and (S)-warfarin. Br. J. Clin. Pharmacol..

[27] Lane S, Al-Zubiedi S, Hatch E (2012). The population pharmacokinetics of R- and S-warfarin: Effect of genetic and clinical factors. Br. J. Clin. Pharmacol..

[28] Martin K, Beyer-Westendorf J, Davidson BL (2016). Use of the direct oral anticoagulants in obese patients: Guidance from the SSC of the ISTH. J. Thromb. Haemost..

[29] Güler E, Babur Güler G, Demir GG (2015). A review of the fixed dose use of new oral anticoagulants in obese patients: Is it really enough?. Anatol. J. Cardiol..

[30] Xu M (2019). Population Pharmacokinetics-Pharmacodynamics Study of Warfarin in Patients with Deep Vein Thrombosis.

[31] Takase T, Ikesue H, Tohi M (2018). Interaction between warfarin and short-term intravenous amiodarone in intensive care unit patients after cardiac surgery. J. Pharm. Health Care Sci..

[32] Santos PC, Marcatto LR, Duarte NE (2015). Development of a pharmacogenetic-based warfarin dosing algorithm and its performance in Brazilian patients: Highlighting the importance of population-specific calibration. Pharmacogenomics.

[33] Albengres E, Le Louët H, Tillement JP (1998). Systemic antifungal agents. Drug interactions of clinical significance. Drug Saf..

[34] Yagi T, Naito T, Kato A (2021). Association between the prothrombin time-international normalized ratio and concomitant use of antibiotics in warfarin users: Focus on type of antibiotic and susceptibility of bacteroides fragilis to antibiotics. Ann. Pharmacother..

[35] Ghaswalla PK, Harpe SE, Tassone D (2012). Warfarin–antibiotic interactions in older adults of an outpatient anticoagulation clinic. Am. J. Geriatr. Pharmacother..

[36] Li Y, Liu H, Lou Y (2015). 1. A comparative study on the accuracy of warfarin stable dose prediction model (Fuhua model 2) in guiding Chinese patients with valvular heart disease replacement surgery. Chin. Circ. J..

[37] Johnson JA, Caudle KE, Gong L (2017). Clinical pharmacogenetics implementation consortium (CPIC) guideline for pharmacogenetics-guided warfarin dosing: 2017 update. Clin. Pharmacol. Ther..

[38] Lee SC, Ng SS, Oldenburg J (2006). Interethnic variability of warfarin maintenance requirement is explained by VKORC1 genotype in an Asian population. Clin. Pharmacol. Ther..

[39] Yuan HY, Chen JJ, Lee MT (2005). A novel functional vkorc1 promoter polymorphism is associated with inter-individual and inter-ethnic differences in warfarin sensitivity. Hum. Mol. Genet..

[40] Yang L, Ge W, Yu F (2010). Impact of VKORC1 gene polymorphism on interindividual and interethnic warfarin dosage requirement–a systematic review and meta analysis. Thromb. Res..

[41] Zhu YB, Hong XH, Wei M (2017). Development of a novel individualized warfarin dose algorithm based on a population pharmacokinetic model with improved prediction accuracy for Chinese patients after heart valve replacement. Acta Pharmacol. Sin..

[42] Shin J, Cao D (2011). Comparison of warfarin pharmacogenetic dosing algorithms in a racially diverse large cohort. Pharmacogenomics.

