# Supplementary material for: Construction of warfarin population pharmacokinetics and pharmacodynamics model in Han population based on Bayesian method
Source: Sci Rep. 2024 Jun 27;14:14846. doi: 10.1038/s41598-024-65048-7 (PMC11211351; doi:10.1038/s41598-024-65048-7)
Supplement: Supplementary file 1 — Supplementary Information. [file 41598_2024_65048_MOESM1_ESM.docx]

**1 Pharmacodynamic analysis of population pharmacokinetics**

1.1 Structural model

Since INR is usually used clinically as the basis for individualized administration of warfarin without the plasma concentration of warfarin, the PK part of the model is simplified to establish the K-PD model (Figure 1). In this model, it is assumed that the drug enters the effect site at the rate of Ke in compartment A (administration chamber), that is, the drug delivery rate at the effect site is DR=A*Ke, where A is the dose of compartment A.

DR together with the parameters E_max_ and EDK_50_ inhibits the formation of activated vitamin K-dependent coagulation factors, and the specific functional relationship can be described by the Sigmoid Emax model:

$\text{E}_{\text{Anticoagulant}}\text{=}\frac{\text{E}_{\text{max}}\text{×}\text{DR}^{\text{γ}}}{\text{EDK}_{\text{50}}^{\text{ }\text{γ}}\text{+}\text{DR}^{\text{γ}}}$ Equation 1

In Equation 1, E _Anticoagulant_ represents the anticoagulant effect after warfarin administration; The E_max_ value is the maximum anticoagulant effect, and this value is fixed at 1, that is, the maximum anticoagulant effect is 100%; EDK_50_ is the DR (drug delivery rate at the effect site) required to achieve 50% of the maximum anticoagulant effect; γ is a shape parameter that reflects the steepness of the change in efficacy with DR value.

Through the exploration of early data modeling, Hamberg finally determined to use 2 transport chamber chains to describe the coagulation effect of different coagulation factors in vivo, each transport chamber chain contains 3 transport chambers, the average transport time (MTT) between each transport chamber in the first transport chamber chain is 29 hours, and the average transport time (MTT) between each transport chamber in the second transport chamber chain is 119 hours.

The anticoagulant effect after warfarin administration affects the coagulation effect of 2 transport chamber chains. The anticoagulant effect is 1 (i.e., 100%) when not used, and 1-E_Anticoagulant_ after warfarin (i.e., E in Figure 1). The coagulation effects of each transport chamber chain after passing through 3 transport chambers are C1_3_ and C2_3_, and the final total coagulation effect is taken as the average of the two, that is (C1_3_+C2_3_)/2, and the index INR of the reaction anticoagulant effect can be expressed as:

$\text{CL=TVCL×[1-(Age-71)×0.00571]}$ Equation 2

In Equation 2, INR _baseline_ is the baseline value of INR. INR _max_ is the maximum value of INR, which is fixed to 20 ^[10]^ according to the literature.（C1_3_+C2_3_）/2 is the coagulation effect (maximum value is 1), and 1-（C1_3_+C2_3_）/2 is the anticoagulant effect. When not used,（C1_3_+C2_3_）/2=1, while INR=INR _baseline_ . When warfarin is used in large doses for a long time,（C1_3_+C2_3_）/2 tends to 0, while INR=INR _baseline_ +INR _max_.


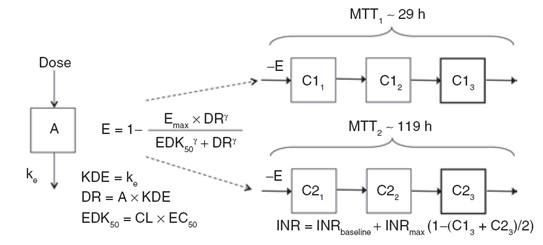


**Figure 1. Schematic diagram of the K-PD structural model** (KDE=CL / V. CL is the drug clearance at the effector site. V is the distribution volume of the drug at the effector site. EC50 is the effect site drug concentration required to reach 50% of the maximum anticoagulant effect. The product of EC50 (unit: mg/L) and CL (unit: L/h) is EDK50 (unit: mg / h))

1.2 Random-effects model

Random-effects models included between-individual variation and intra-individual variation. For inter-individual variation, the literature model introduces inter-individual variation for the parameters KDE (i.e., ke) and EC_50_, using an exponential model. Since the model parameters of pharmacokinetics and pharmacodynamics in most cases conform to the lognormal distribution, that is, they conform to the exponential model, the new model will still use the exponential model to describe the inter-individual variation of the model parameters (KDE and EC_50_).

Exponential model: $\text{P}_{\text{i}}\text{=}\text{P}_{\text{tot}}\text{*}\exp\text{(}\text{η}_{\text{i}}\text{)}$ Equation 3

In Equation 3, P_i_ is the model parameter value of the i-th subject, P _tot_ is the population parameter value, and η_i_ is the inter-individual variation of the i-th subject model parameter, which corresponds to the normal distribution with a mean of 0 and a variance of ω^2^.

For intra-individual variation, the literature model adopts a proportional residual model. In addition to the proportional residual model, the new model in this study will also examine the additive model and the proportional addition model.

Scale model: $\text{IN}\text{R}_{\text{obs,ij}}\text{=IN}\text{R}_{\text{pred,ij}}\text{*(1+}\text{ε}_{\text{ij,prop}}\text{)}$ Equation 4

Addition model: $\text{IN}\text{R}_{\text{obs,ij}}\text{=IN}\text{R}_{\text{pred,ij}}\text{+}\text{ε}_{\text{ij,add}}$ Equation 5

Proportional addition model: $\text{IN}\text{R}_{\text{obs,ij}}\text{=IN}\text{R}_{\text{pred,ij}}\text{*(1+}\text{ε}_{\text{ij,prop}}\text{)+}\text{ε}_{\text{ij,add}}$ Equation 6

In Equations 4 to 6，INR_obs,ij_ and INR_pred,ij_ are the INR observations and predicted values at the j-th time point of the i-th subject. ε_ij, prop_ and ε_ij,add_ are proportional and additive individual residual variants at the j-th time point of the i-th subject, corresponding to the normal distribution of mean 0 and variance σ_1_^2^ and σ_1_^2^, respectively.

1.3 Covariate model

A covariate model is built to filter for covariates that affect structural model parameters. The literature model introduces CYP2C9 genotype and age as covariates into the model parameter CL, and VKORC1 genotype as a covariate into the model parameter EC50, while age is a continuous covariate, which is introduced into CL using a scale model (Equation 7).

$\text{CL=TVCL×[1-(Age-71)×0.00571]}$ Equation 7

In Equation 7, TVCL is the typical population value of CL, and the model expression shows that for every 1 year of age, the CL value decreases by 0.571%.

The two strands of the CYP2C9 gene contain three phenotypes, *1, *2 and *3, and the CL values of 0.174 L/h, 0.0879 L/h and 0.0422 L/h are defined by the literature model, respectively. Therefore, the CL values of different genotypes of CYP2C9 are:

*CYP2C9* **1*/**1*：0.174+0.174=0.348 L/h

*CYP2C9* **1*/**2*：0.174+0.0879=0.2619 L/h

*CYP2C9* **1*/**3*：0.174+0.0422=0.2162 L/h

*CYP2C9* **2*/**1*：0.0879+0.174=0.2619 L/h

*CYP2C9* **2*/**2*：0.0879+0.0879=0.1758 L/h

*CYP2C9* **2or*3*：0.0879+0.0422=0.1301 L/h

*CYP2C9* **3*/**1*：0.0422+0.174=0.2162 L/h

*CYP2C9* **3*/**2*：0.0422+0.0879=0.1301 L/h

*CYP2C9* **3*/**3*：0.0422+0.0422=0.0844 L/h

The two strands of the VKORC1 gene contain two phenotypes, G and A, and the literature model defines their corresponding EC_50_ values of 1.55 mg/L and 0.782 mg/L, respectively. Therefore, the EC_50_ values for different genotypes of VKORC1 are:

*VKORC1* *AA*：0.782+0.782=1.564 mg/L

*VKORC1* *AG*：0.782+1.55=2.332 mg/L

*VKORC1* *GA*：1.55+0.782=2.332 mg/L

*VKORC1* *GG*：1.55+1.55=3.1 mg/L

Since the analysis data do not contain the PK information of warfarin, the model parameters of the PK part of this article are directly fixed as the parameter estimation values of the literature model. The model parameter EC_50_ values of the PD part are re-estimated according to the data characteristics of the Han Chinese population.

In addition, in addition to CYP2C9 genotype, VKORC1 genotype and age, this article will also examine the effects of weight, body mass index, sex, amiodarone, azole antimicrobials, statins, and broad-spectrum antimicrobials on the EC_50_ values of the model parameters. The continuous covariate is represented by a power function model (Equation 8), while Piece-wise model for categorical variables (Equation 9)

$P_{i}\text{=}\text{P}_{\text{TV}}\text{×(}\frac{\text{Cov}}{\text{Cov}_{\text{median}}}\text{)\textasciicircumθ}$ Equation 8

$\text{P}_{\text{i}}\text{=}\left\{ \begin{aligned} \text{P}_{\text{TV}}\text{ if COV=0} \\ \text{P}_{\text{TV}}\text{×}\left( \text{1+}\text{} \right)\text{ if COV=1} \end{aligned} \right.$ Equation 9

P_TV_ is the population typical value of the parameter. θ is the correction factor of the individual parameter. COV is the covariate value, and COV median is the median number. If a subject's continuous covariate is missing, the median of the covariate is filled. If a subject's categorical covariate is missing, the classification with the highest frequency is filled.

1.4 Covariate screening methods

Firstly, the graphical method is used to analyze the correlation between the covariates to be investigated and the EC_50_ values of the model parameters, and the significantly related covariates (P<0.05) are screened. The covariates that are significantly related to the model parameters are added to the structural model according to the models described in Equations 8 and 9, respectively. If the objective function value of the model decreases by more than 6.63 (the cut-off value of P=0.01 of the chi-square distribution with 1 degrees of freedom), it indicates that the covariate has a significant influence on the model parameters. The covariates with significant influencing factors are screened by the forward method and the backward method, and the covariates that finally entered the model are confirmed, and the Alfalfa-Warfarin-PPK/PD model is established. The OFV cut-off for the forward method is set at 6.63 (P<0.01) and the backward method is set at 7.78 (P<0.005).

1.5 Model evaluation methods

After the Alfalfa-Warfarin-PPK/PD model is established, the performance of the model needs to be evaluated. Firstly, on the modeling dataset, the goodness-of-fit of the proposed model is evaluated by diagnostic graph method, and the goodness-of-fit of the literature model is compared. Secondly, on the validation dataset, the goodness-of-fit of the proposed model is evaluated by diagnostic graph method, and the goodness-of-fit of the literature model is compared. In this way, the advantages of the proposed model over the literature model are evaluated. Model diagnostic plots include Observation (OBS) and Population Prediction (PRED) and Individual Predictions (IPRED) scatterplots, Conditional-Weighted Residuals (CWRES) and time and PED scatter plots. In addition, the median and 95% confidence interval of the model parameter distribution are obtained by 1000 retractable repeated sampling by the Bootstrap method, and the model parameters obtained from the original dataset were compared with the model parameters obtained in the original dataset, so as to evaluate the stability of the model parameters.

1.6 Simulation

According to the Alfalfa-Warfarin-PPK/PD model, the mutual estimation of doses or INR values at any moment is simulated. Typical INR values after stabilization at different doses are simulated at different covariate levels(Dose 0.125mg to 7.5mg once a day), and the dose of warfarin corresponding to the INR in the target range of 2 to 3 at steady state (when the risk of bleeding and thromboembolism is lowest ^[4]^) is calculated to provide recommendations for individualized administration.

During the simulation, the baseline INR value was fixed at 1.13, which was the mean INR of the subjects in the total data set. When one covariate was examined, the other covariables were fixed as typical values, and the typical values of each covariate were CYP2C9 genotype *1/*1, VKORC1 genotype AA, unincorporated amiodarone, body weight 60kg, and age 55 years.

The results showed that after homeostatic INR of typical subjects reached 2, 2.5 and 3, the corresponding warfarin administration doses were 2.5mg, 3.5mg and 4.625mg, respectively. When CYP2C9 genotype is *1/*3, the above INR target values should be reached. The doses of warfarin were reduced to 1.875mg, 2.75mg and 3.625mg, respectively. With VKORC1 genotype AG, warfarin dose would need to be increased from 2.5mg to 5.625mg to achieve INR 2, and warfarin dose would need to be increased by more than 7.5mg (beyond the simulated dose range) to achieve INR 2.5 or 3. Similarly, when VKORC1 genotype was GG, INR reached the standard and higher doses were required. The steady-state value of INR at 7.5mg of warfarin could not reach the target value of 2. When combined with amiodarone, the dosage of warfarin should be reduced to 1.875mg, 2.625mg and 3.5mg for the target INR of 2, 2.5 and 3, respectively. The warfarin dose should be increased when the body weight of the subjects increased, and the warfarin dose corresponding to the INR of 2, 2.5 and 3 for 80kg subjects was increased to 3.5mg, 5.125mg and 6.625mg, respectively. For 40kg body weight subjects with INR target of 2, 2.5 and 3, warfarin doses were reduced to 1.5mg, 2.125mg and 2.875mg, respectively. The warfarin dose should be reduced as the age of the subjects increases, and the warfarin dose corresponding to the INR target of 2, 2.5, and 3 for 70-year-old subjects should be reduced to 2.25mg, 3.25mg, and 4.25mg, respectively. The warfarin dose was increased to 2.625mg, 3.75mg, and 5mg for 40-year-old subjects with an INR target of 2, 2.5, and 3, respectively.

| *CYP2C9* | *VKORC1* |
| --- | --- |
| 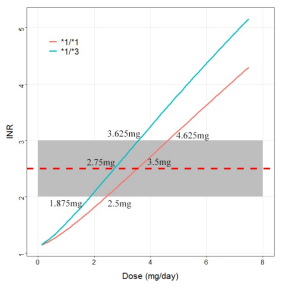 | 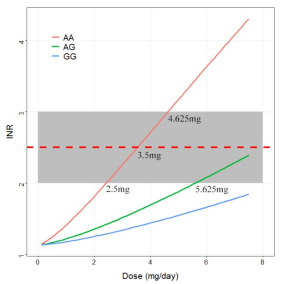 |
| CM1 | Weight |
| 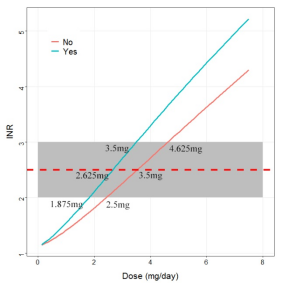 | 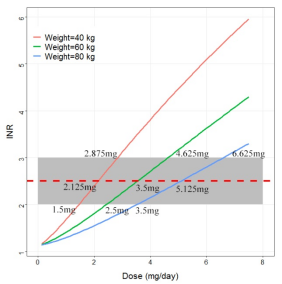 |
| *Ag*e |  |
| 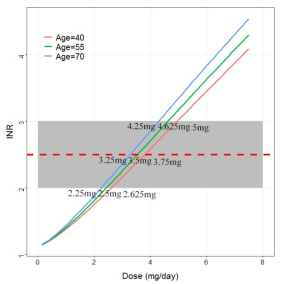 |  |

**Figure 2. The typical value distribution of INR in subjects with different covariates at different doses after reaching stability(**CM1 represents whether amiodarone is incorporated.The gray shaded bands in the figure are the target interval 2-3 of INR, and the red interval represents the target value of 2.5. The doses indicated in the figure are the warfarin dose corresponding to patients achieving INR target values (2,2.5 and 3) at different covariate levels).


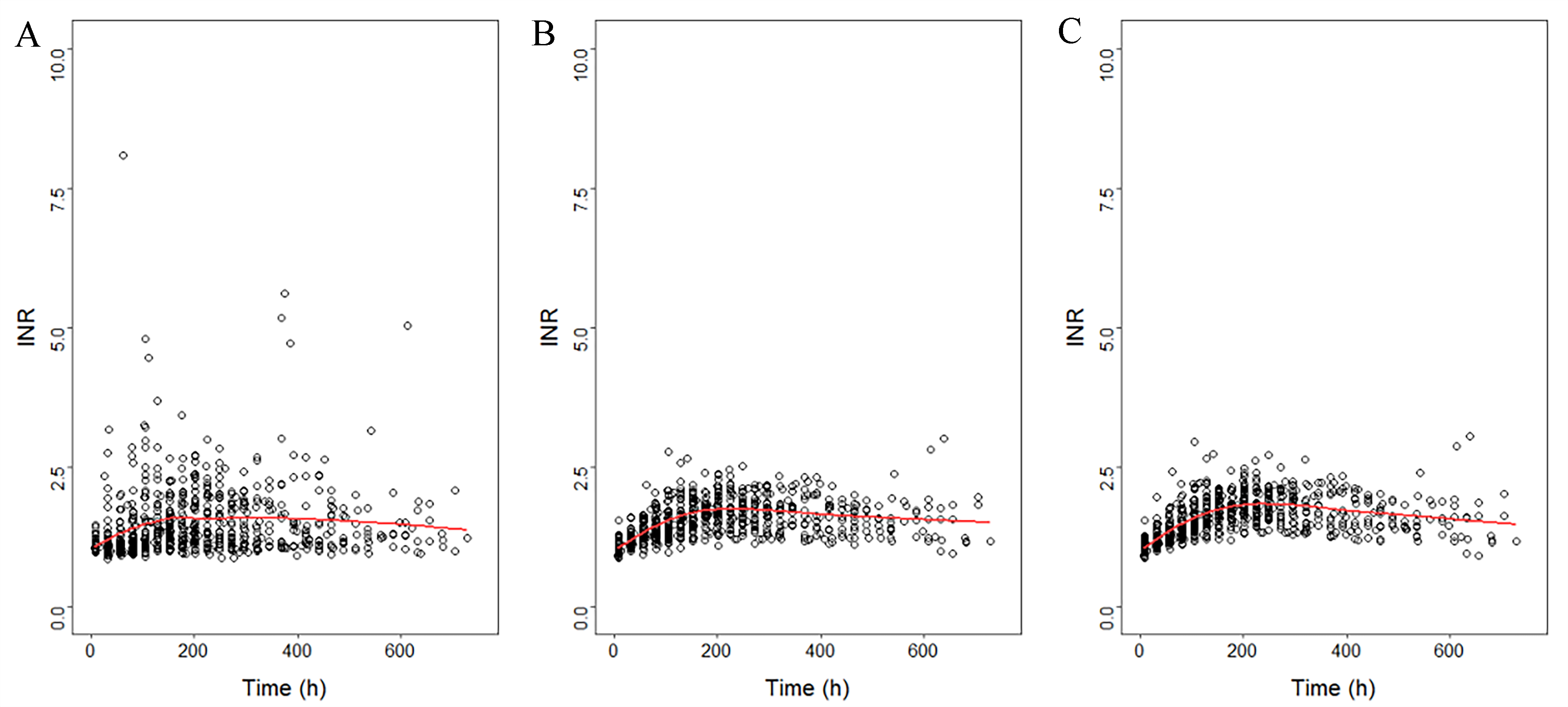


**Figure 3. Distribution of INR measured value and Alfalfa-Warfarin-PPK/PD model predicted value** (The scatter points in the figure are the measured INR value and the model predicted value, and the red solid line is the trend line of the INR distribution. A: INR Measured value, B: Alfalfa-Warfarin-PPK/PD Model - INR Predicted Values, C: Literature Model - INR Predicted Values).


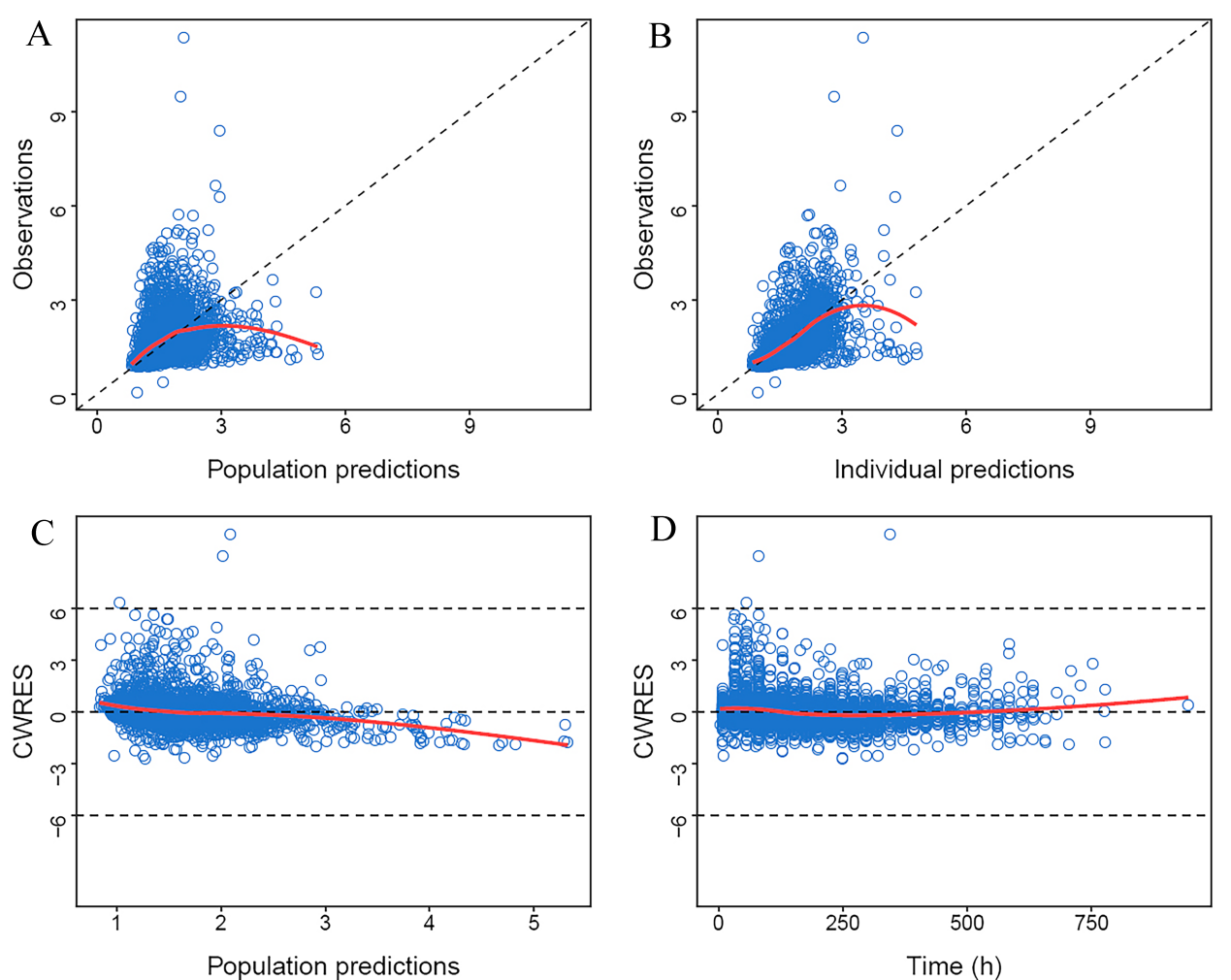


**Figure 4. Final model diagnostic diagram** (A scatter plot of observed value vs group predicted value, B scatter plot of observed value vs individual predicted value, the dashed line in the figure is the accuracy (diagonal line), and the red solid line is the fitted line. C scatter plot of conditional weighted error vs population prediction value, D scatter plot of conditional weighted error vs blood collection time point, the red solid line is the trend line.).


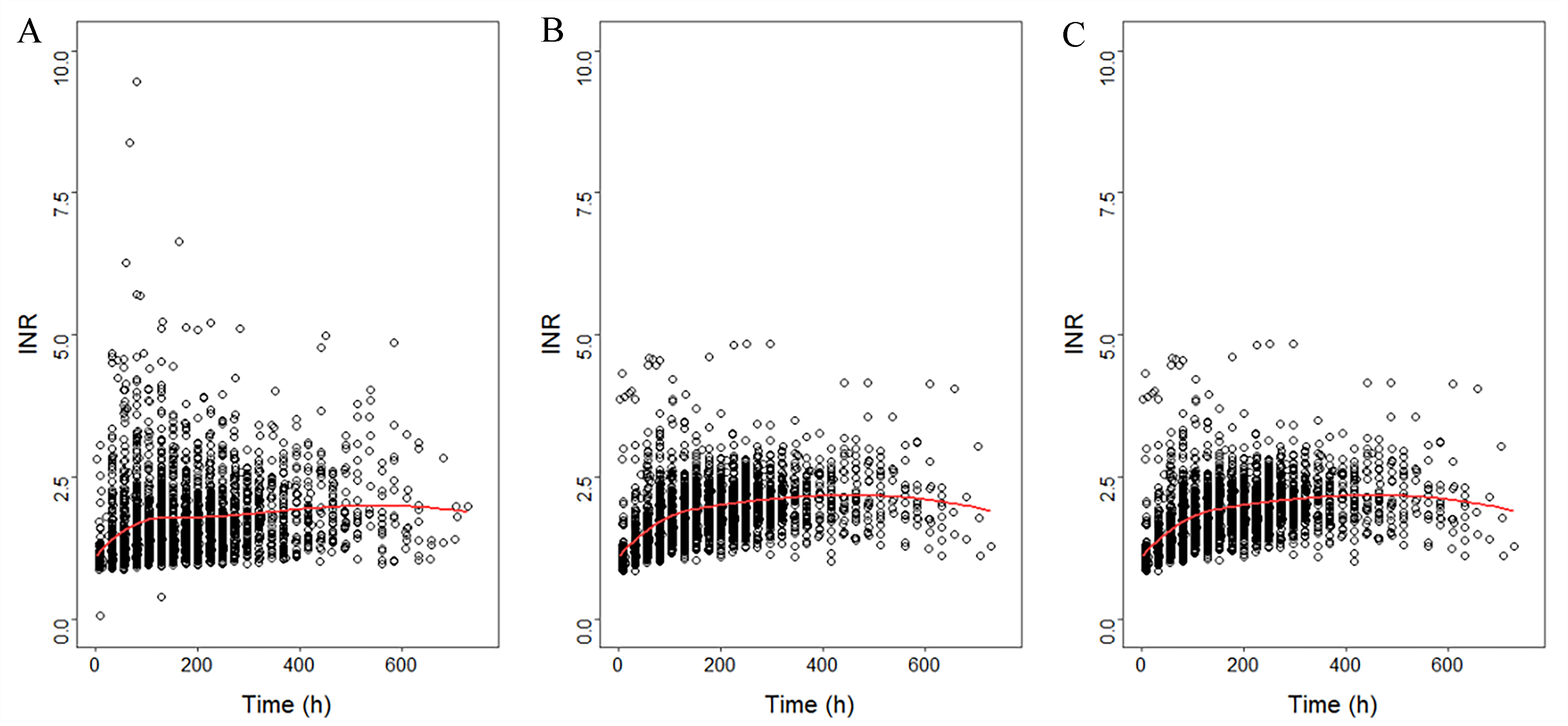


**Figure 5. Distribution of INR measured values and Alfalfa-Warfarin-PPK/PD model predicted values in the validation dataset** (The scatter points in the figure are the measured INR value and the model predicted value, and the red solid line is the trend line of the INR distribution. A: INR Measured value, B: Alfalfa-Warfarin-PPK/PD Model - INR Predicted Values, C: Literature Model - INR Predicted Values)


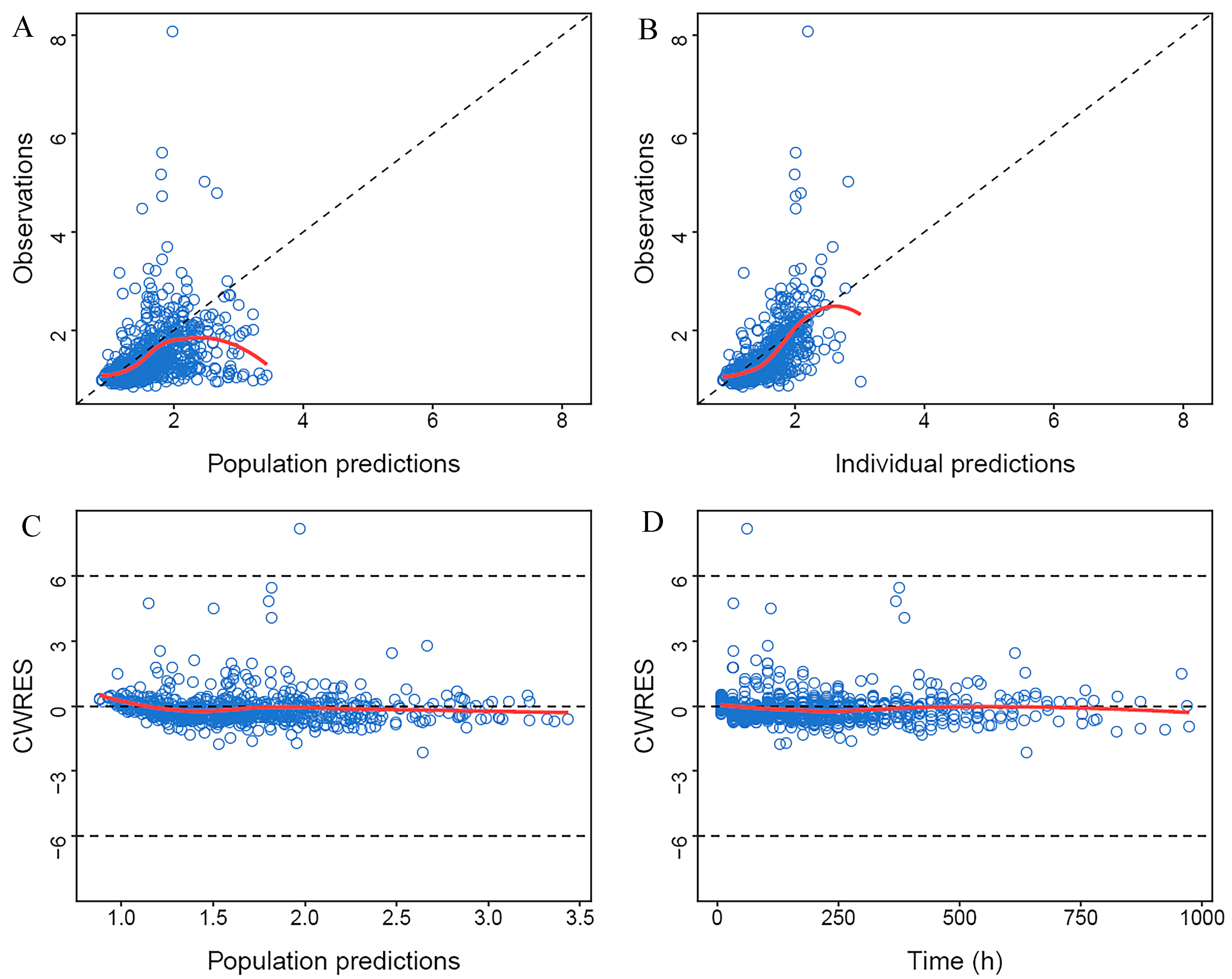


**Figure 6. Model diagnostic diagram of the Alfalfa-Warfarin-PPK/PD model on the validation dataset** (A scatter plot of observed value vs group predicted value, B scatter plot of observed value vs individual predicted value, the dashed line in the figure is the accuracy (diagonal line), and the red solid line is the fitted line. C scatter plot of conditional weighted error vs population prediction value, D scatter plot of conditional weighted error vs blood collection time point, the red solid line is the trend line.).


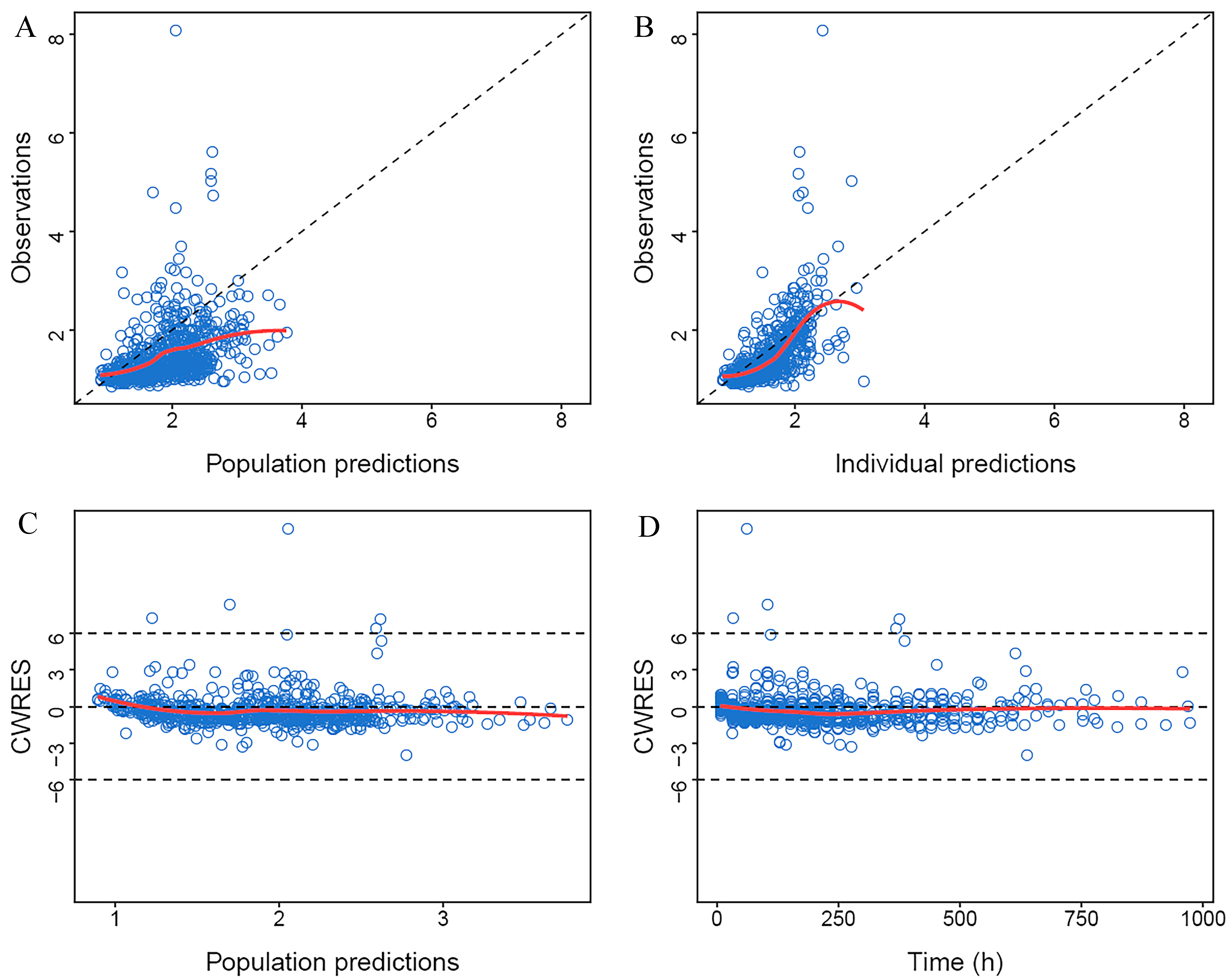


**Figure 7. Model diagnosis diagram of the literature model on the validation dataset** (A scatter plot of observed value vs group predicted value, B scatter plot of observed value vs individual predicted value, the dashed line in the figure is the accuracy (diagonal line), and the red solid line is the fitted line. C scatter plot of conditional weighted error vs population prediction value, D scatter plot of conditional weighted error vs blood collection time point, the red solid line is the trend line.).

**Table 1 Details of the included models**

|  | Gage Model^[12]^ | IWPC Model ^[13]^ | Hamberg Model ^[11]^ |
| --- | --- | --- | --- |
| Year | 2008 | 2009 | 2010 |
| Modeling / model testing | 1015/292 | 4043/1009 | 1015/292 |
| Race | 82.5% Caucasian/15.1% African American/2.4% Other | 56% Caucasian/30% Asian/10% African American/5% Other | 29% Italy / 71% Sweden |
| Model parameters | Race, age, body surface area, smoking status, venous thrombosis, *VKORC1*, *CYP2C9*, amiodarone | Race, Age, Height, Weight, *VKORC1*, *CYP2C9*, Enzyme Inducer, Amiodarone | Age, base INR, *CYP2C9*, *VKORC1* |
| Modeling method | MAR | MAR | MAPB |
| R^2^ | 53.1 | 47.0 | 53.1 |

**Table 2 Correlation comparison between the prediction warfarin dose and the actual dose of each model**

|  | Mean±SD | R | P |
| --- | --- | --- | --- |
| Actual dose | 2.698±0.849 | 1 |  |
| Alfalfa-Warfarin-PPK/PD Model | 3.088±1.466 | 0.767 | <0.001 |
| Hamberg Model | 3.126±0.764 | 0.72 | <0.001 |
| IWPC Model | 4.193±0.582 | 0.607 | <0.001 |
| Gage Model | 3.428±0.711 | 0.466 | <0.001 |
| FDA Model | 3.516±0.895 | 0.444 | <0.001 |

**Table 3 MPE comparison of each model**

| Model | Mean | SD |
| --- | --- | --- |
| Alfalfa-Warfarin-PPK/PD Model | 0.390 | 0.019 |
| Hamberg Model | 0.427 | 0.018 |
| FDA Model | 0.818 | 0.027 |
| Gage Model | 0.924 | 0.017 |
| IWPC Model | 1.495 | 0.020 |

**Table 4 Comparison of prediction percentage of each model**

| Model | Prediction dose <80% | Prediction dose 80-120% | Prediction dose >120% |
| --- | --- | --- | --- |
| Alfalfa-Warfarin-PPK/PD Model | 36（3.06）^*^ | 856（72.60）^**^ | 287（24.34）^***^ |
| Hamberg Model | 46（3.90） | 603（51.15） | 530（44.95） |
| FDA Model | 78（6.62） | 530（44.95） | 571（48.43） |
| Gage Model | 61（5.17） | 576（48.85） | 542（45.97） |
| IWPC Model | 6（0.50） | 561（47.5） | 621（51.91） |

Note: The total number of researchers validated by each model is 1179. * indicates the total number and percentage of patients whose predicted dose is less than 80% of the actual dose in the study; ** Indicates the total number and percentage of patients in the study whose predicted dose was 80% greater than the actual dose and less than 120% of the actual dose; indicates the total number and percentage of patients in the study where the predicted dose is greater than 120% of the actual dose;
